# NGSpeciesID: DNA barcode and amplicon consensus generation from long‐read sequencing data

**DOI:** 10.1002/ece3.7146

**Published:** 2021-01-11

**Authors:** Kristoffer Sahlin, Marisa C. W. Lim, Stefan Prost

**Affiliations:** ^1^ Department of Mathematics Science for Life Laboratory Stockholm University Stockholm Sweden; ^2^ Department of Population Health and Reproduction University of California Davis CA USA; ^3^ LOEWE‐Centre for Translational Biodiversity Genomics Senckenberg Frankfurt Germany; ^4^ South African National Biodiversity Institute National Zoological Garden Pretoria South Africa

**Keywords:** amplicon sequencing, DNA barcoding, sequence clustering, third‐generation sequencing

## Abstract

Third‐generation sequencing technologies, such as Oxford Nanopore Technologies (ONT) and Pacific Biosciences (PacBio), have gained popularity over the last years. These platforms can generate millions of long‐read sequences. This is not only advantageous for genome sequencing projects, but also advantageous for amplicon‐based high‐throughput sequencing experiments, such as DNA barcoding. However, the relatively high error rates associated with these technologies still pose challenges for generating high‐quality consensus sequences. Here, we present NGSpeciesID, a program which can generate highly accurate consensus sequences from long‐read amplicon sequencing technologies, including ONT and PacBio. The tool includes clustering of the reads to help filter out contaminants or reads with high error rates and employs polishing strategies specific to the appropriate sequencing platform. We show that NGSpeciesID produces consensus sequences with improved usability by minimizing preprocessing and software installation and scalability by enabling rapid processing of hundreds to thousands of samples, while maintaining similar consensus accuracy as current pipelines.

## Graphical abstract

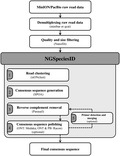

## 1 INTRODUCTION

We are in the middle of a biodiversity crisis, in which anthropogenic change is driving many species to extinction, often faster than they can be characterized (see, e.g., Ceballos et al., 2020). The identification of species in our environments is paramount to informing conservation policy and practice. The development of DNA barcoding (Hebert et al., 2003) was a major step toward large‐scale characterizations of biodiversity. This technique utilizes amplification of standardized genetic regions to characterize species present within biological samples. Besides the documentation of biodiversity, this method and other amplicon sequencing technologies have been widely used for monitoring of invasive species, detection of pathogens in environmental samples, and many other applications in taxonomy, medicine, or evolutionary biology (e.g., reviewed in Kress et al., 2015).

Third‐generation sequencing is able to sequence millions of single molecules up to several Mbs in lengths (Jain et al., 2018). Currently, two platforms are readily available for DNA barcoding efforts, PacBio's Sequel II and ONT’s MinION. These platforms offer the advantage of longer reads, at the cost of sequencing errors. While ONT’s MinION still shows higher error rates >5% (Wick et al., 2018), the new PacBio HiFi mode allows for the generation of read with <1% error (Wenger et al., 2019), which will greatly improve the generation of accurate DNA barcodes. Early on, researchers identified the potential of third‐generation sequencing platforms for sequencing much longer DNA barcodes than previously possible (see, e.g., Tedersoo et al., 2018; Krehenwinkel, Pomerantz, Henderson, et al., 2019; Wurzbacher et al., 2019). Beside the longer amplicon length, ONT’s MinION also offers the advantage that sequencing can be carried out almost anywhere in the world, due to its small size and affordability (reviewed in Krehenwinkel, Pomerantz, & Prost, 2019). While there has been a considerable software development effort to assemble high‐quality amplicon consensus sequences from error‐prone ONT MinION reads (see, e.g., Maestri et al., 2019; Seah et al., 2020; Srivathsan et al., 2019; reviewed in Krehenwinkel, Pomerantz, & Prost, 2019), only a few software solutions are available for PacBio‐based DNA barcodes (see, e.g., Wurzbacher et al., 2019). To our knowledge, of these, only the pipeline presented in Wurzbacher et al. (2019) is able to handle both PacBio and ONT sequencing reads.

Here, we present NGSpeciesID a one‐software solution for reconstructing high‐quality amplicon consensus sequences for both PacBio and ONT sequencing reads. We also investigate the performance of ONT’s Medaka polishing software compared to Racon (Vaser et al., 2017) for MinION‐based DNA barcoding. Compared to other programs, NGSpeciesID can be easily installed with conda, does not require any specific file name structures, can handle data from both third‐generation sequencing types, includes different consensus polishing options, and only needs fastq files as input. We show that our tool produces consensus sequences of a similar quality than other software solutions, while reducing the burden to users by requiring little to no additional tools or data reformatting.

## 2 SOFTWARE DESCRIPTION

**FIGURE 1:**
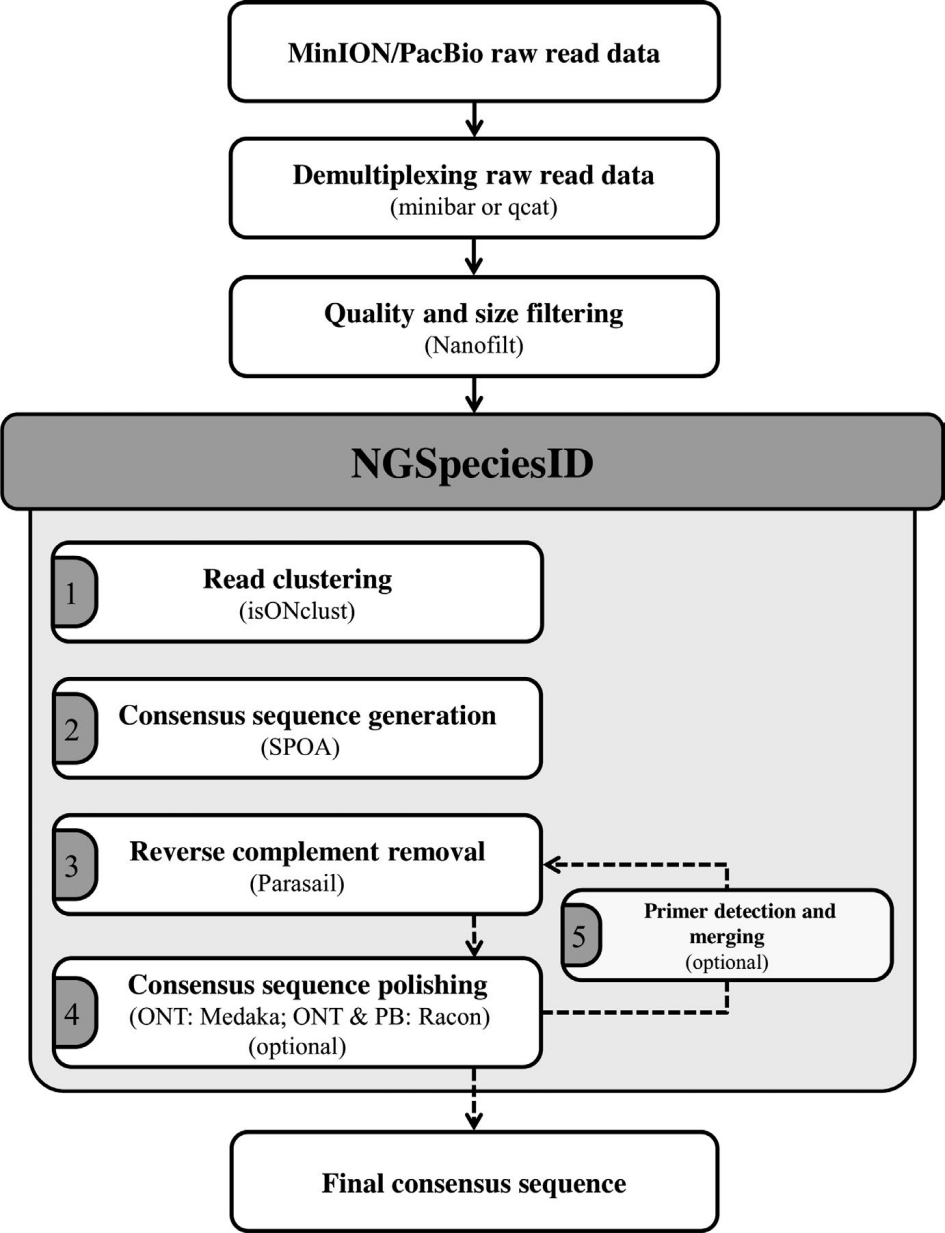
Steps involved in DNA barcode consensus calling of long‐read data. The respective software tools used in the different steps are provided in brackets. In the first step, long‐read data are usually demultiplexed. After demultiplexing, the reads are filtered for read length and quality. This step can also be carried out before demultiplexing if the respective amplicons do not differ in length. Next, consensus sequences for the individual read files can be generated using NGSpeciesID. If multiple read files need to be processed, NGSpeciesID can be run in a pipeline (see File S14). Within the tool, reads are first clustered according to similarity. Next, consensus sequences are generated for each cluster larger than an abundance threshold (default: >10% of all reads). In the third step, NGSpeciesID checks the generated consensus sequences for reverse complementary. If consensus sequences are reverse complement, then the respective clusters are merged. In step four, the consensus sequences are polished using the reads from the respective clusters (this step is optional). In the last optional step, primers can be removed if this was not already carried out by the demultiplexing or basecalling tools. If primers are removed, NGSpeciesID will carry out steps 3–4 again

NGSpeciesID is a program developed in python that wraps a set of tools for read clustering, consensus forming, and consensus polishing (Figure 1). It is a one‐software solution and extension of the Saiga pipeline, we developed previously (Seah et al., 2020). It can be easily installed using the free and open‐source Anaconda distribution. Briefly, NGSpeciesID clusters amplicon sequencing reads (in fastq format) and forms a consensus sequence for each cluster. Next, it merges reverse‐complement clusters. Finally, the remaining consensus sequence(s) is/are polished. Optionally, the tool can also remove primer sequences from the consensus after the polishing step. In the following sections, we describe the workflow of NGSpeciesID, which is freely available at https://github.com/ksahlin/NGSpeciesID. For more details, see File S1 and Figure 1.

### 2.1 Clustering of reads

NGSpeciesID first clusters input sequence reads based on expected sequence similarity for ONT or PacBio reads. Clustering is performed to remove any sequencing artifacts or contamination and assures that only similar reads from the relevant amplicon region will be considered when producing a polished amplicon. NGSpeciesID uses the isONclust clustering algorithm, which accounts for variable error rates within reads and is designed for both ONT or PacBio sequencing technologies. isONclust was recently shown to perform better than other clustering algorithms on both ONT or PacBio data (Sahlin & Medvedev, 2019).

### 2.2 Forming draft consensus

Next, a draft consensus amplicon sequence is formed for each cluster that contains more reads than a specified proportion of the total reads (default: 10% of the total number of reads). The draft consensus sequences are formed with spoa (Vaser et al., 2017).

### 2.3 Reverse‐complement detection and removal

During the clustering step, reverse‐complement reads from the amplicon region can produce two separate clusters of the same amplicon. In order to assure only one sequence per amplicon, NGSpeciesID detects and merges any consensus sequences classified as reverse‐complement sequences with respect to each other using pairwise alignment with parasail (Daily, 2016). To do so, all consensus sequences are aligned to each other and any two sequences with alignment identity over a parameter (default: 90%) are merged. The original reads that were used to generate the two consensus sequences are combined to increase coverage. Finally, all draft consensus sequences passing this step, with the original reads, are sent to the polishing step.

### 2.4 Polishing

The remaining consensus sequences are polished with either Medaka (https://github.com/nanoporetech/medaka) or Racon (Vaser et al., 2017). In this step, the original reads are first mapped back to the consensus sequence (reference sequence). The reference is then corrected using sequence information from the multiple reads mapped. The polished consensus sequences are the final output of NGSpeciesID.

### 2.5 Primer detection and removal

Many basecalling and demultiplexing tools do not remove primers from the amplicon sequences (but see Minibar (Krehenwinkel, Pomerantz, Henderson, et al., 2019)). NGSpeciesID, therefore, implements an optional primer removal step by searching the forward and reverse complement of each primer within a window at each end of the read. This step is carried out for the polished sequences to improve the detection of the priming sites. If no primer is found, the polished consensus sequence(s) remain the final output of NGSpeciesID. If primer(s) have been detected and trimmed, NGSpeciesID reruns the reverse‐complement removal and polishing steps to identify any remaining redundant consensus sequences that were not removed due to primers.

## 3 USE CASES AND COMPARISON TO OTHER TOOLS

We tested our software on publicly available data from Maestri et al. (2019) and Wurzbacher et al. (2019), and compared the accuracy of respective consensus sequences generated in the two studies to those reconstructed with NGSpeciesID. To measure accuracy, we aligned the consensus sequences to the respective Sanger sequence using BLAST (Altschul et al., 1990) and calculated accuracy as the sum of all matches in the alignment divided by the alignment length. We chose the two software solution (Mothur (Schloss et al., 2009) and Consension (Wurzbacher et al., 2019)) presented in Wurzbacher et al. (2019) for our comparison as it is currently, to our knowledge, the only one that can be used with both PacBio and ONT sequencing reads. We further compared our result to the ONTrack software (Maestri et al., 2019) developed for ONT data specifically. In both comparisons, we carried out polishing within NGSpeciesID using Medaka (https://github.com/nanoporetech/Medaka) and Racon (Vaser et al., 2017).

### 3.1 Comparison to Mothur + Consension

We randomly selected five out of the 61 fungi datasets from Wurzbacher et al. (2019), ranging from 201 to 447 reads per dataset (Table S1). These cover five fungi species of the genus *Inocybe* for ribosomal DNA (rDNA) and the full ribosomal tandem repeat region (TR). We provide alignments of the corresponding Sanger sequences with our consensus sequences in the Files S2–S6). In their approach, Wurzbacher et al. (2019) first perform operational taxonomic unit (OTU) clustering on the read data using Mothur (Schloss et al., 2009). Next, they create consensus sequences using Consension (Wurzbacher et al., 2019).

In general, we see that for both ONT and PacBio data NGSpeciesID and the Mothur + Consension software perform equally well, generating consensus sequences with 98.6% to 100% accuracy (Table 1). In three out of the five cases, the two pipelines produced consensus sequences with the same accuracy, while in one case each software slightly outperformed the other (Table 1). Medaka polishing outperformed Racon polishing in four out of five cases (Table 1).

**TABLE 1:** Percent similarity to the respective Sanger sequence for the datasets 17075, 17078, 16416, 16427, and 16483 from Wurzbacher et al. (2019)

SampleID	17075	17078	16416	16427	16483
NGSpeciesID					
ONT Medaka	**98.6% (10/726)**	**99.2% (6/741)**	99.5% (4/731)	**99.6% (3/790)**	**100% (0/709)**
ONT Racon	98.5% (11/726)	99.1% (7/741)	99.7% (2/731)	99.1% (7/790)	99.9% (1/709)
PB Racon	**98.6% (10/726)**	99.1% (7/741)	**99.9% (1/731)**	**99.6% (3/790)**	**100% (0/709)**
Mothur + Consension					
ONT	**98.6% (10/726)**	**99.2% (6/741)**	**99.9% (1/731)**	99.5% (4/790)	**100% (0/709)**
PB	**98.6% (10/726)**	**99.2% (6/741)**	99.7% (2/731)	**99.6% (3/790)**	**100% (0/709)**

Note

The highest similarity scores are highlighted in bold. The numbers in the brackets provide the amount of mismatches to the Sanger reference and the length of the reference sequence.

### 3.2 Comparison to ONTrack

Next, we compared the performance of NGSpeciesID to the pipeline ONTrack from Maestri et al. (2019). ONTrack first clusters all reads using VSEARCH (Rognes et al., 2016), then randomly selects 200 reads, aligns those with Mafft (Katoh & Standley, 2013), calls the consensus with EMBOSS cons (http://emboss.sourceforge.net/apps/cvs/emboss/apps/cons.html), and lastly carries out polishing with 200 randomly selected reads using Nanopolish (https://github.com/jts/nanopolish.). We generated consensus sequences for all seven DNA barcodes from Maestri et al. (2019), which comprise *cytochrome C oxidase subunit 1* (COI) sequences of two snails and five beetles (Table S1). We provide the respective alignments in the Files S7–S13).

Previously, Krehenwinkel, Pomerantz, Henderson, et al. (2019) showed that consensus accuracy can decrease when too many reads (in the realm of a few hundred reads, depending on the error rate of the individual reads) are selected for the consensus generation, likely due to an increase in the signal to noise ratio. We thus randomly subsampled 300 reads using seqtk (command: *seqtk sample ‐s 1234 reads.fastq 300 > reads_subsample.fastq*; https://github.com/lh3/seqtk), a number which has been shown to work well with Nanopore data (Krehenwinkel, Pomerantz, Henderson, et al., 2019). We see that the consensus quality is comparable between the two tools (Table 2), with accuracy of 99.8% to 100%. In five out of the seven DNA barcode sets, both tools performed equally well, while in one each the two tools outperformed each other, however, differing by only 1 base pair (Table 2).

**TABLE 2:** Percent similarity to the respective Sanger sequence for the datasets B1 to BC7 from Maestri et al. (2019)

SampleID	BC1^a^	BC2	BC3	BC4^a^	BC5	BC6^a^	BC7^a^
NGSpeciesID							
ONT Medaka	**100% (0/651)**	**100% (0/658)**	99.9% (1/649)	**100% (0/606)**	**100% (0/658)**	**99.8% (1/576)**	**100% (0/536)**
ONT Racon	99.5% (3/651)	99.5% (3/658)	98.9% (7/649)	99.2% (5/606)	99.8% (1/658)	99.7% (2/576)	99.4% (3/536)
ONTrack							
ONT	99.9% (1/651)	**100% (1** ^b^ **/658)**	**100% (2** ^b^ **/649)**	**100% (0/606)**	**100% (2** ^b^ **/658)**	**99.8% (1/576)**	**100% (0/536)**
Mixed							
NGSpeciesID							
ONT Medaka	**100% (0/651)**	**100% (0/658)**	99.7% (2/649)	99.3% (4/606)	**100% (0/658)**	**99.8% (1/576)**	99.6% (2/536)

Note

For the mixed samples, 300 reads of each of the seven DNA barcodes were combined into a single file, from which NGSpeciesID generated multiple consensus sequences. NGSpeciesID was run using Medaka polishing.

^a^ Here, the Sanger sequence from Maestri et al. (2019) was shorter than the expected fragment length and all the consensus sequences. In these cases, we only calculated the percentage similarity for the region covered by the respective Sanger sequence.

^b^ The consensus sequences from Maestri et al. (2019) are missing one or two bases at the start, which could be due to a consensus calling error, or deletion of one additional base during the primer removal. For the percentage accuracy, we assumed them to be incorrectly trimmed. The highest similarity scores are highlighted in bold. The numbers in the brackets provide the amount of mismatches to the Sanger reference and the length of the reference sequence.

### 3.3 Mixed samples

We tested NGSpeciesID’s performance on mixed samples in silico by combining 300 reads of each of the seven barcodes from Maestri et al. (2019). To do so, we set the cluster abundance ratio to 5% (‐‐abundance_ratio 0.05). We recovered seven consensus sequences corresponding to the seven DNA barcodes, ranging from 99.3% to 100% similarity to the corresponding Sanger sequence (Table 2). In four out of the seven cases, we recovered the same percentage similarity to the Sanger sequence in the mixed analysis as in the respective single barcode processing. In three cases, the accuracy was slightly lower with two and four basepair differences, respectively.

## 4 DISCUSSION

### 4.1 Consensus quality

Here, we present NGSpeciesID, an easy‐to‐use, one‐software solution for the generation of high‐quality consensus sequences for the long‐read sequencing technologies from ONT and PacBio. We compared NGSpeciesID against results obtained with Mothur + Consension and ONTrack. In general, all three software solutions produced consensus sequences of a very high quality, reaching 99%–100% accuracy in almost all cases. We show that NGSpeciesID performs comparably to the other tools. Throughout all comparisons, we see that consensus sequences based on ONT data polished with Racon usually show lower percent similarities to the Sanger sequence than consensus sequences polished with Medaka. NGSpeciesID carries out 2 rounds of Racon polishing by default. Increasing or decreasing the number of rounds might increase the consensus quality. We chose Medaka as the default error corrector in NGSpeciesID as it includes up to date error models. We did not include an option to use Nanopolish in NGSpeciesID, which is used in ONTrack, as this tool requires fast5 files, which are often not available for published Oxford Nanopore data. Furthermore, it requires preprocessing to generate the appropriate header structure in the corresponding fastq files, which makes it much more time consuming to use.

As the generation of consensus sequences for DNA barcoding takes only a few seconds for each sample (depending on the number of reads), we did not compare run times between the different pipelines.

### 4.2 Easy use

NGSpeciesID was designed to be straightforward to use. It works on individual read files, outputted either directly from the basecalling or after demultiplexing (e.g., using Minibar (Krehenwinkel, Pomerantz, Henderson, et al., 2019) or qcat (https://github.com/nanoporetech/qcat)), but can quickly be adjusted to run in a loop over multiple fastq files using a bash script (see File S14). It only requires fastq files as input. In contrast, ONTrack requires the input reads in three formats (fast5, fasta and fastq), which requires additional preprocessing of the sequencing data. Furthermore, NGSpeciesID allows fastq files to have any naming structure, thus making it easy for the user to run and to identify samples and replicates. This saves time on preprocessing of the read data compared to other software solutions.

NGSpeciesID employs quality filtering of the reads based on read phred scores. However, we recommend also removing reads much shorter or longer than the intended target, which often represent chimeras or contaminations using NanoFilt (De Coster et al., 2018) before running NGSpeciesID. While our tool can handle unfiltered data, this might result in the generation of multiple consensus sequences. NGSpeciesID also offers the option to remove priming sites from the amplicon sequences. As many universal primers include ambiguity codes, primer regions can potentially include incorrect bases and should thus be removed. We further found that primer regions can cause issues for the reverse‐complement matching. We thus included an additional reverse‐complement matching step after primer removal, in case NGSpeciesID outputs multiple consensus sequences. Our tool outputs multiple consensus sequences in case the clustering results in multiple clusters over a certain percentage of the total reads (by default this is set to 10%). Each consensus sequence is only polished with the corresponding reads from the clustering. This feature is very useful as it allows the user to explore potential contaminant reads or mixed samples through the generating of multiple consensus sequences.

NGSpeciesID and the Mothur + Consension software solution both can handle ONT and PacBio long‐read data. While both tools produce consensus sequences of similar accuracy, Mothur + Consension requires an in‐depth knowledge of the pipeline requiring (a) preprocessing of the input data, (b) individual components of the pipeline to be run separately, and (c) has parameter settings that are difficult to interpret, while NGSpecies is designed to be user friendly and packaged as a one command solution.

### 4.3 Mixed samples

While NGSpeciesID was not designed specifically for metabarcoding data, the flexibility of the algorithmic steps in the pipeline enables the tool to handle mixed samples if they are sufficiently divergent. We recovered seven consensus sequences corresponding to the seven DNA barcodes pooled in the mixed sample analysis. NGSpeciesID generated highly accurate consensus sequences for all barcodes, ranging from 99.2% to 100%. For the mixed sample test, we adjusted the read abundance ratio for the clusters to 5%, since the seven barcodes at equal abundance are each present in only 14% of the reads in the sample. Therefore, the default abundance cutoff of 10% would require 210 out of the 300 reads to be used per cluster, which might not be the case. Three out of seven barcodes showed a slightly lower consensus accuracy than in the respective single species analysis, which is likely due to the presence of some reads from other barcodes in the clusters that might have affected the polishing accuracy, and the random selection of the 300 reads for each barcode (as individual read error rates can differ). We expect some cross‐contamination (reads assigned to the wrong cluster), especially for closely related species. However, this should improve with the continued improvement of third‐generation sequencing read accuracy. This experiment shows that NGSpeciesID, even though it was not developed for mixed samples, can recover highly accurate consensus sequences from metabarcoding data if the samples are sufficiently divergent. However, its performance on metabarcoding data will need to be investigated separately with mock datasets of varying ratios and sample relationships (to see which taxonomic divergences are needed for effective separation of reads from related species).

## 5 CONCLUSION AND FUTURE DIRECTIONS

We present NGSpeciesID, an easy‐to‐use and flexible one‐software solution to generate high‐quality consensus sequences for both ONT and PacBio sequencing data. It performs equally well as other pipelines and software solutions tested here, but offers advanced usability as it is simple to use and does not require preprocessing of the data before running. Portable devices such as the inexpensive MinION sequencer have started to democratize the process of molecular biodiversity monitoring (see, e.g., Krehenwinkel, Pomerantz, and Prost (2019). Here, we add to this, by the development of a simple to install and run bioinformatic software that should further enable students and citizen scientists without a formalized bioinformatic training to carry out biodiversity monitoring and assessment studies.

## CONFLICT OF INTEREST

The authors have declared that no competing interests exist.

## AUTHOR CONTRIBUTION

**Kristoffer Sahlin:** Conceptualization (Equal), Software (Equal), Writing‐original draft (Equal), Writing‐review & editing (Equal) **Marisa C. W. Lim:** Conceptualization (equal); Software (equal); Writing‐original draft (equal); Writing‐review & editing (equal). **Stefan Prost:** Conceptualization (Lead), Methodology (Equal), Project administration (Lead), Software (Equal), Supervision (Lead), Writing‐original draft (Lead), Writing‐review & editing (Lead)

## ETHICS STATEMENT

The presented study only used publicly available data.

## Supporting information

Supplementary Materials

## Data Availability

We did not generate sequencing read data within this study. GenBank accession numbers for all samples used in this study along with the citations of the papers they were published in are provided in Table S1. The software along with example read data can be found on https://github.com/ksahlin/NGSpeciesID.
