# Supplementary material for: NGSpeciesID: DNA barcode and amplicon consensus generation from long‐read sequencing data
Source: Ecol Evol. 2021 Jan 11;11(3):1392–8. doi: 10.1002/ece3.7146 (PMC7863402; doi:10.1002/ece3.7146)
Supplement: Supplementary file 1 — Supplementary Materials [file ECE3-11-1392-s001.zip › ece37146-sup-0001-FileS1.docx]

**Software description**

***Clustering of reads***

NGSpeciesID first clusters the reads based on expected sequence similarity for ONT or PacBio reads. The clustering algorithm used in NGSpeciesID is the isONclust algorithm which is described in detail in Sahlin and Medvedev (2019). In brief, before the clustering step, reads are sorted with respect to expected number of error-free kmers in descending order. The expected number of error-free kmers in a read is predicted based on the quality (phred scores) values and the length of the read. The reads are then traversed in descending order starting with the read with the most expected error-free kmers first. A read is either added as a new *cluster representative* (forming a new cluster), or *matched* to a previously created cluster representative based on the sequence similarity between the read and the representative. To quickly compute whether a read matches a representative, isONclust computes and stores minimizers (Roberts et al., 2004) in a database for each cluster representative. The number of shared minimizers between a read and a representative can be quickly inferred from the minimizer database. The number of shared minimizers as well as their positions across the read is then used to decide whether the read is inferred to come from the same amplicon as the representative. The requirements for a match are selected dynamically based on the read quality of both the read and the representative. In the event that a read shares minimizers with several representatives, the representative with the most shared minimizers is considered first in the matching step. If no match was found for a read, isONclust has the option to fall back on a more time-consuming but sensitive exact alignment step using parasail (Daily, 2016). After all reads are traversed, they are either themselves a representative of a cluster, or part of a cluster. Within NGSpeciesID, isONclust is used with default parameters.

***Forming draft consensus***

Next, a draft consensus is formed for each cluster that contains more reads than an abundance threshold (default: 10% of the total number of reads). The draft consensus sequences are formed with spoa (Vaser et al., 2017), applying the non-default parameter -g 2 which penalizes deletions less than the default setting.

***Reverse complement detection and removal***

NGSpeciesID then detects and merges any consensus sequences classified as reverse complement sequences using pairwise alignment with parasail (Daily, 2016). All consensus sequences are aligned to the reverse complements of the other sequences. Two consensus sequences are merged if they have a sequence identity above a parameter given to NGSpeciesID (default 10%). Sequence identity is calculated as 1 - mismatches/alignment-length, where mismatches can be either indels or substitutions. If one or more of the consensus sequences are classified as reverse complements to each other, the consensus from the larger cluster is kept as the reference, and the reads from the smaller cluster are combined with the consensus from the larger cluster. Finally, all draft consensus sequences passing this step, together with the original reads, are sent to polishing. The number of reads from all combined clusters are provided in the fasta sequence header of the consensus sequences.

***Polishing***

The remaining consensus sequences are polished with either Medaka (<https://github.com/nanoporetech/medaka>) or Racon (Vaser et al., 2017). If Medaka is specified as the polishing algorithm, a training model parameter can be specified to allow a suitable model choice for the sequence protocol from which the data was generated. The default model protocol parameter is “r941_min_high_g330”, and can be adjusted according to the MinION flow cell version. If Racon is specified as the polishing algorithm, a parameter can be set to allow Racon to perform iterative polishing that can improve consensus quality (default: 2 iterations). The polished consensus sequences are the final output of NGSpeciesID.

***Primer detection and removal***

Many basecalling and de-multiplexing tools do not remove primers from the DNA barcodes (but see Minibar (Krehenwinkel et al., 2019)). NGSpeciesID, therefore, implements an optional primer removal step by searching the forward and reverse complement of each primer (provided in a fasta file) within a window at each end of the read. This is carried out for the polished sequences. If no primer is found, the polished consensus sequence(s) remain the final output of NGSpeciesID. If primer(s) have been detected and trimmed, NGSpeciesID reruns the reverse-complement removal and polishing steps to identify any remaining redundant consensus sequences that were not removed due to primers.

The primer removal is implemented as follows. Let c denote the length of the consensus sequence and let x be an integer provided as a parameter to the program with a default value of 150. The window length for each end is set to min(x, c/2). The adapter sequences are searched for using edlib (Šošić et al. 2017). A hit of a primer sequence to the consensus is defined as a location on the consensus that has edit distance at most y between the primer and the consensus, where y is a parameter to NGSpeciesID with (default value of 2). In the case that there are several optimal hits (i.e., having the same smallest edit distance) of the primer within the window, the innermost hit with respect to the consensus is considered and the consensus sequence is trimmed at this point.

***Differences to the SAIGA pipeline***

We previously released SAIGA (Seah et al. 2020), which has similar underlying softwares. However, NGSpeciesID offers many improvements compared to SAIGA. 1) NGSpeciesID is able to handle both ONT and PacBio long-read data, 2) it offers a streamlined software installation, 3) we have replaced cd-hit (<http://weizhongli-lab.org/cd-hit/>) with parasail (Daily 2016), which is much more efficient in detecting reverse complement consensus sequences, 4) NGSpeciesID includes filtering options such as filtering based on average PHRED quality scores, 5) we provide both Medaka (<https://github.com/nanoporetech/medaka>) and Racon (Vaser et al., 2017) as consensus polishing options, and lastly 6) NGSpeciesID is able to detect and remove PCR priming sites.

**References**

Daily, J. (2016). Parasail: SIMD C library for global, semi-global, and local pairwise sequence alignments. *BMC bioinformatics*, *17*(1), 81.

Krehenwinkel, H., Pomerantz, A., Henderson, J.B., Kennedy, S.R., Lim, J.Y., Swamy, V., Shoobridge, J.D., Graham, N., Patel, N.H., Gillespie, R.G., Prost, S., 2019a. Nanopore sequencing of long ribosomal DNA amplicons enables portable and simple biodiversity assessments with high phylogenetic resolution across broad taxonomic scale. Gigascience 8. https://doi.org/10.1093/gigascience/giz006

Sahlin, K., & Medvedev, P. (2020). De Novo Clustering of Long-Read Transcriptome Data Using a Greedy, Quality Value-Based Algorithm. *Journal of Computational Biology*, *27*(4), 472-484.

s

Seah, A., Lim, M.C.W., McAloose, D., Prost, S., Seimon, T.A., 2020. MinION-Based DNA Barcoding of Preserved and Non-Invasively Collected Wildlife Samples. Genes 11, 445. https://doi.org/10.3390/genes11040445

Šošić, M. and Šikić, M. (2017). Edlib: a C/C ++ library for fast, exact sequence alignment using edit distance. Bioinformatics btw753. doi: 10.1093/bioinformatics/btw753

Vaser, R., Sović, I., Nagarajan, N. & Šikić, M. Fast and accurate de novo genome assembly from long uncorrected reads. Genome Res. 27, 737–746 (2017).
