# Supplementary material for: NGSpeciesID: DNA barcode and amplicon consensus generation from long‐read sequencing data
Source: Ecol Evol. 2021 Jan 11;11(3):1392–8. doi: 10.1002/ece3.7146 (PMC7863402; doi:10.1002/ece3.7146)
Supplement: Supplementary file 1 — Supplementary Materials [file ECE3-11-1392-s001.zip › ece37146-sup-0014-FileS14.pdf]

##Bash script to run NGSpeciesID on multiple samples automatically

##Additional parameter such as removing primers, etc. can easily be added to the command

##This bash script will create consensus sequences for all fastq files in the working directory.
#All corresponding folders will have the same base name as the respective fastq files.

##To run the script save it as a file, eg. NGSpeciesID.sh and make it executable with “chmod
#755 NGSpeciesID.sh” and then run in the working directory with ./NGSpeciesID.sh

```
for file in *.fastq; do
	bn=`basename $file .fastq`
	NGSpeciesID --ont --consensus --medaka --fastq $file --outfolder ${bn}
done
```
